# Monitoring and manipulating cellular crosstalk during kidney fibrosis inside a 3D *in vitro* co-culture

**DOI:** 10.1038/s41598-017-12683-y

**Published:** 2017-11-03

**Authors:** Bramasta Nugraha, Manuel A. Mohr, Aaron Ponti, Maximilian Y. Emmert, Franziska Weibel, Simon P. Hoerstrup, Solange Moll, Ulrich Certa, Marco Prunotto, Periklis Pantazis

**Affiliations:** 1 Department of Biosystems Science and Engineering (D-BSSE), Eidgenössische Technische Hochschule (ETH) Zurich, Mattenstrasse 26, 4058 Basel, Switzerland; 2 Roche Pharmaceutical Research and Early Development (pRED), Roche Innovation Center Basel, 4070 Basel, Switzerland; 3 Institute for Regenerative Medicine (IREM), University of Zurich, Wagistrasse 12, 8952 Schlieren, Switzerland; 4 Department of Surgical Research, University Hospital Zurich, Rämistrasse 100, 8091 Zurich, Switzerland; 5 University Heart Center, University Hospital Zurich, Rämistrasse 100, 8091 Zurich, Switzerland; 6 Wyss Translational Center Zurich, Moussonstrasse 13, 8044 Zurich, Switzerland; 7 Department of Pathology, University Hospital of Geneva, Geneva, Switzerland; 8 Present Address: Institute for Regenerative Medicine (IREM), Wyss Translational Center Zurich, University of Zurich, Wagistrasse 12, 8952, Schlieren, Switzerland

## Abstract

In pharmacological research the development of promising lead compounds requires a detailed understanding of the dynamics of disease progression. However, for many diseases, such as kidney fibrosis, gaining such understanding requires complex real-time, multi-dimensional analysis of diseased and healthy tissue. To allow for such studies with increased throughput we established a dextran hydrogel-based *in vitro* 3D co-culture as a disease model for kidney fibrosis aimed at the discovery of compounds modulating the epithelial/mesenchymal crosstalk. This platform mimics a simplified pathological renal microenvironment at the interface between tubular epithelial cells and surrounding quiescent fibroblasts. We combined this 3D technology with epithelial reporter cell lines expressing fluorescent biomarkers in order to visualize pathophysiological cell state changes resulting from toxin-mediated chemical injury. Epithelial cell damage onset was robustly detected by image-based monitoring, and injured epithelial spheroids induced myofibroblast differentiation of co-cultured quiescent human fibroblasts. The presented 3D co-culture system therefore provides a unique model system for screening of novel therapeutic molecules capable to interfere and modulate the dialogue between epithelial and mesenchymal cells.

## Introduction

The discovery of pharmaceutical lead compounds able to treat human disease effectively requires a thorough understanding of its molecular and genetic basis. Such molecular information is essential to identify therapeutic targets that can be used to source the drug discovery process^1,2^. To this end, access to clinical samples can advance the pathological, molecular, and prognostic characterization of an illness. However, accessibility to human samples is limited - and in many cases biased - by the very specific stage of the disease at which such tissues are typically collected for diagnostic analyses^3^.

Animal models have been proposed as an alternative or as a complementation to human tissue samples, yet they are often limited in their ability to effectively mimic human disorders^4,5^. Notably, only a handful of successful insights generated by those *in vivo* models have in fact been translated into the clinical scenario^6,7^ and later into clinical practice^8,9^. To better recapitulate key pathological events, emphasis can instead be placed on human cell-derived *in vitro* culture systems. Conventional two-dimensional (2D) cultures can exhibit differentiated cell functions, yet they commonly fail to mimic tissue- and organ-level structures and functions that are central to disease etiology^10,11^. Thus, there is an immediate need for alternative experimental systems that can recapitulate the tissue- and organ-level pathophysiology *in vitro*.

Like traditional 2D-cell culture, three-dimensional (3D) *in vitro* cell culture technology shares the advantages of being accessible to microscopic examination, chemical intervention, and biological manipulation. They have the added benefit of closer resembling physiological tissue organization and are therefore a powerful tool to investigate fundamental questions regarding morphogenesis, physiology, and disease dynamics^12–16^.

Kidney nephrons, exhibiting a complex physiological structure, have posed challenges for traditional *in vitro* cell culture experimentation^17^. The immense clinical relevance of human kidney fibrosis has recently led to the development of different *in vitro* human microtissue models to simulate the kidney nephron^18,19^ and to target such models for drug-induced toxicity^20,21^.

Damaged polar epithelial cells of the kidney proximal tubular epithelial compartment typically characterize kidney fibrosis. Their degradation is accompanied by an increase in activated myofibroblasts in the interstitial space, which in turn results in continuous accumulation of extracellular matrix and the progressive decline of renal function. In the past years, several lines of evidence have consistently highlighted the role of epithelial cells in controlling the myofibroblast phenotype^22–26^, with some studies demonstrating a major role of tubular injury in the etiology of fibrosis. This experimental indication is supported by extensive clinical evidence that severity of acute kidney injury, mainly consisting of an epithelial cell injury, predicts progression to chronic kidney disease, characterized by active myofibroblast proliferation^27,28^. Despite the availability of several *in vitro* human microtissue models, developed essentially to investigate drug-induced renal toxicity^20,21^, none of those models has allowed direct monitoring of epithelial to mesenchymal crosstalk.

Here, we report a novel 3D *in vitro* cell co-culture model closely resembling the *in vivo* human kidney microenvironment. Using live cell imaging we show the essential role of tubular cells in inducing the phenotypic transformation of the co-cultured fibroblasts into myofibroblasts. In our model, clinically relevant nephrotoxic drugs were used to induce a diseased epithelial phenotype, which in turn triggered transformation of co-cultured fibroblasts, a relevant pathophysiological phenomenon observed *in vivo* ^29,30^ and in patients^31^. Finally, we show that healthy renal fibroblast appearance is successfully rescued by drug inhibition of known fibrosis pathways. The presented 3D co-culture therefore provides a unique model system for screening of new molecules capable to interfere and modulate the dialogue between epithelial and mesenchymal cells.

## Results

### Developing an *in vitro* 3D human renal microenvironment

**Figure 1:**
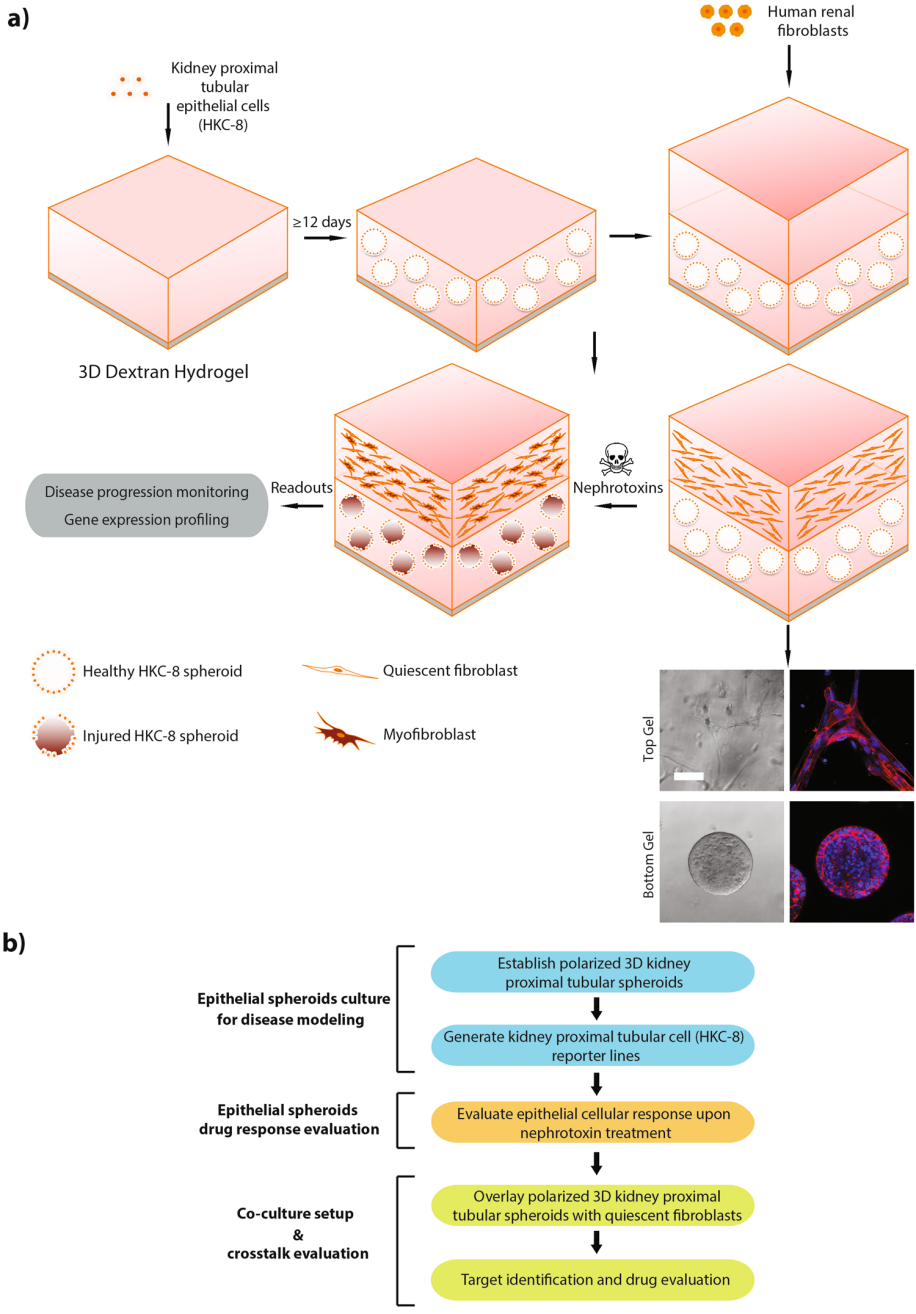
Building up an *in vitro* 3D human renal microenvironment. (**a**) Schematic stepwise illustration of the proposed *in vitro* 3D co-culture system composed of human kidney proximal tubular cells (HKC-8) and human renal fibroblasts: HKC-8 cells self-organize into spheroids within 12 days post seeding. Subsequently overlaid fibroblasts spread throughout the upper gel. Bottom panel: bright field images (left panel) and F-actin immunostaining (right panel; red, F-actin; blue, DAPI) of the two different cell types seeded in hydrogel. Bottom layer: HKC-8 spheroids; upper layer: spreading fibroblast. (**b**) Workflow of the proposed *in vitro* 3D co-culture system. Scale bar, 100 μm (**a**).

**Figure 2:**
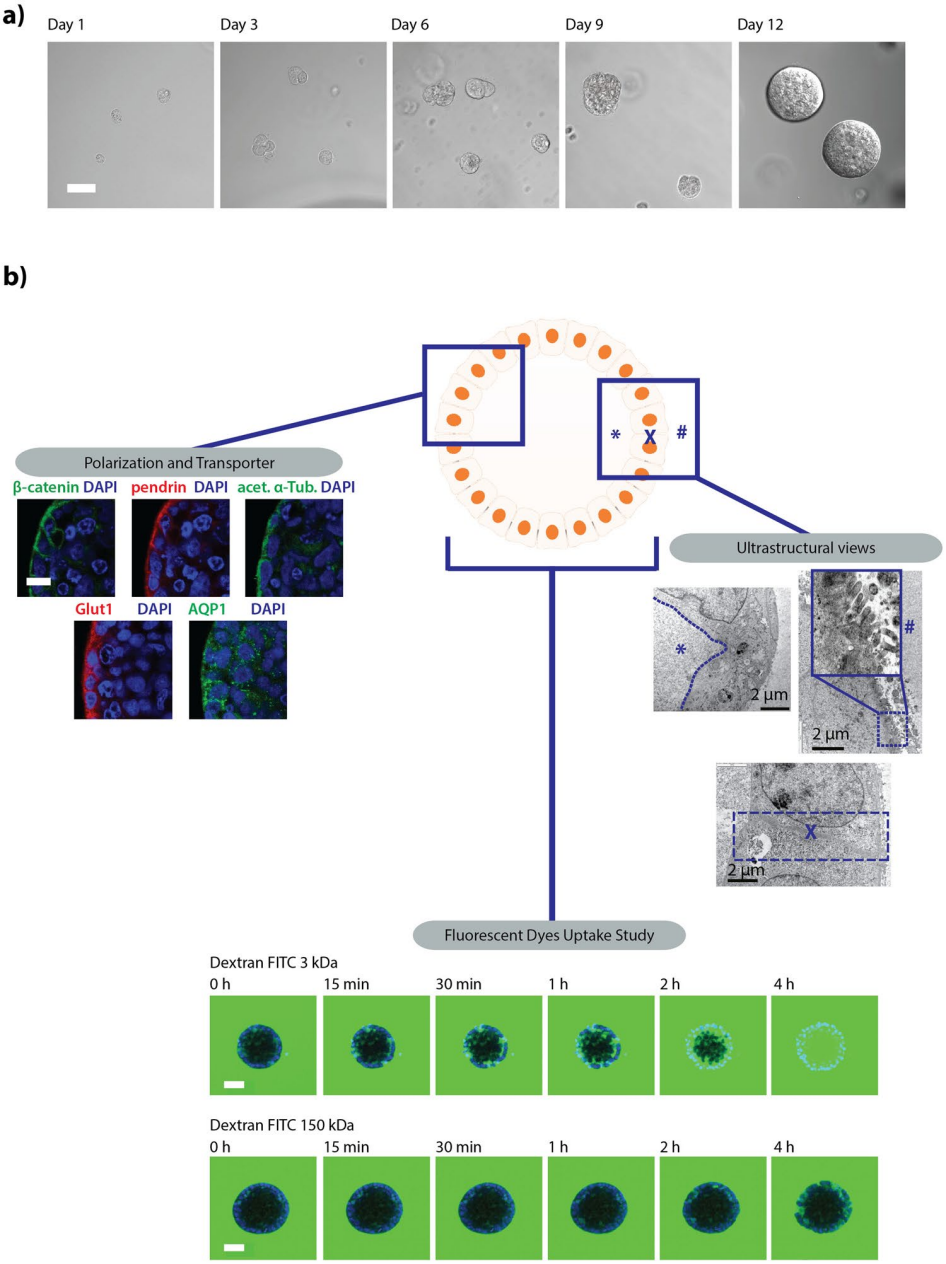
Human kidney proximal tubular (HKC-8) cells form 3D spheroids with distinct epithelial polarity. (**a**) Bright field images of single cell suspension HKC-8 cells seeded in dextran hydrogel forming HKC-8 spheroids after 12 days. (**b**) Functional characterization of HKC-8 spheroids: left panel: Immunofluorescent stainings. right panel: ultrastructural view of the HKC-8 spheroids using transmission electron microscopy (TEM): spheroids display a lumen (left upper panel), presence of microvilli (right upper panel), and tight junction between adjacent cells (lower panel) (*, X, and # indicate hollow lumen structure, cell-cell tight junction, and microvilli, respectively). Bottom HKC-8 spheroid passive molecular uptake study with fluorescently-labeled dextran low molecular weight (3 kDa, upper panel) and high molecular weight (150 kDa, lower panel). Scale bars, 50 μm (**a**), 10 μm (**b**, left), 2 µm (**b**, right), and 50 μm (**b**, bottom).

We set out to create a platform that would conveniently allow simultaneous monitoring of epithelial cell injury and induction of the fibroblast to myofibroblast transition in co-cultured fibroblasts ultimately enabling imaging-based drug screens (Fig. 1a and b). To this end, we developed a stepwise *in vitro* 3D co-culture system made up of two layers of polyethylene glycol (PEG)-crosslinked dextran hydrogels, encompassing the different constituent cell phenotypes: The bottom layer contains polarized human kidney tubular cell (HKC-8) spheroids whereas the top layer holds quiescent primary human renal fibroblasts (Fig. 1a). Upon sparse seeding in dextran hydrogel the HKC-8 cells gathered to form cell aggregates. After 6 to 9 days, lumen formation in the spheroid core commenced and by day 12, the majority of structures contained a central hollow lumen surrounded by a uniform layer of columnar epithelium (Fig. 2a). On day 15, the hydrogel was overlaid with a second one containing MMP-sensitive peptide crosslinker to facilitate fibroblasts spreading and migration (Fig. 1a). This stepwise approach allowed for differential fine-tuning of the two separate microenvironments, ensuring optimal cell differentiation for both cell phenotypes. This solution was adopted as the tubular cells in the bottom compartment require a hydrogel that prevents cell spreading to maintain differentiated function, whereas the fibroblasts in the top layer require cellular cues for cell spreading and migration to develop their characteristic phenotype.

### HKC-8 spheroids recapitulate essential architectural characteristics relevant to the human renal tubular compartment

In order to test the quality of our established 3D cell culture system, we assessed mature HKC-8 spheroids for polarity, ultrastructure, and permeability. At 15 days post-seeding, HKC-8 spheroids recapitulated essential architectural characteristics relevant to the human renal tubular compartment, such as polarized localization of β-catenin to adherent junctions and to basolateral membranes as well as presence of polarized ciliated cell surfaces (Fig. 2b). As expected for functional renal tubules, the spheroids expressed several renal transporters, such as aquaporin water channel 1 (AQP1), glucose transporter 1 (GLUT1), and sodium-independent chloride/iodide transporter (Pendrin), all being enriched at the cell surface (Fig. 2b left hand side of the figure). Ultrastructural analysis of the spheroids performed by transmission electron microscopy (TEM) corroborated features detected via immunofluorescent staining: tight junctions were present between neighboring columnar renal epithelial cells, which enclosed a hollow lumen and possessed outward extending primary cilia (Fig. 2b on the right). Consistent with our structural analysis, tubular cell spheroids exhibited tissue-specific tight barriers, necessary for water reabsorption (Fig. 2b on the bottom)^32^. When FITC-conjugated dextran with molecular weight (MW) below the reported MW cutoff of proximal tubules (3 kDa) was added to the culture medium, it gradually filled the spheroid lumen within 4 hours of incubation time. Higher molecular weight FITC-conjugated dextran (150 kDa) on the other hand was excluded from the lumen and remained restricted to apical intercellular regions, indicating that the spheroids exhibit physiological renal reabsorption capabilities. These data suggest that HKC-8 spheroids can be generated in soft dextran hydrogel, retaining all main features of differentiated tubular epithelial cells.

### The nephrotoxic compounds aristolochic acid and cyclosporine A can induce significant HKC-8 spheroids injury

**Figure 3:**
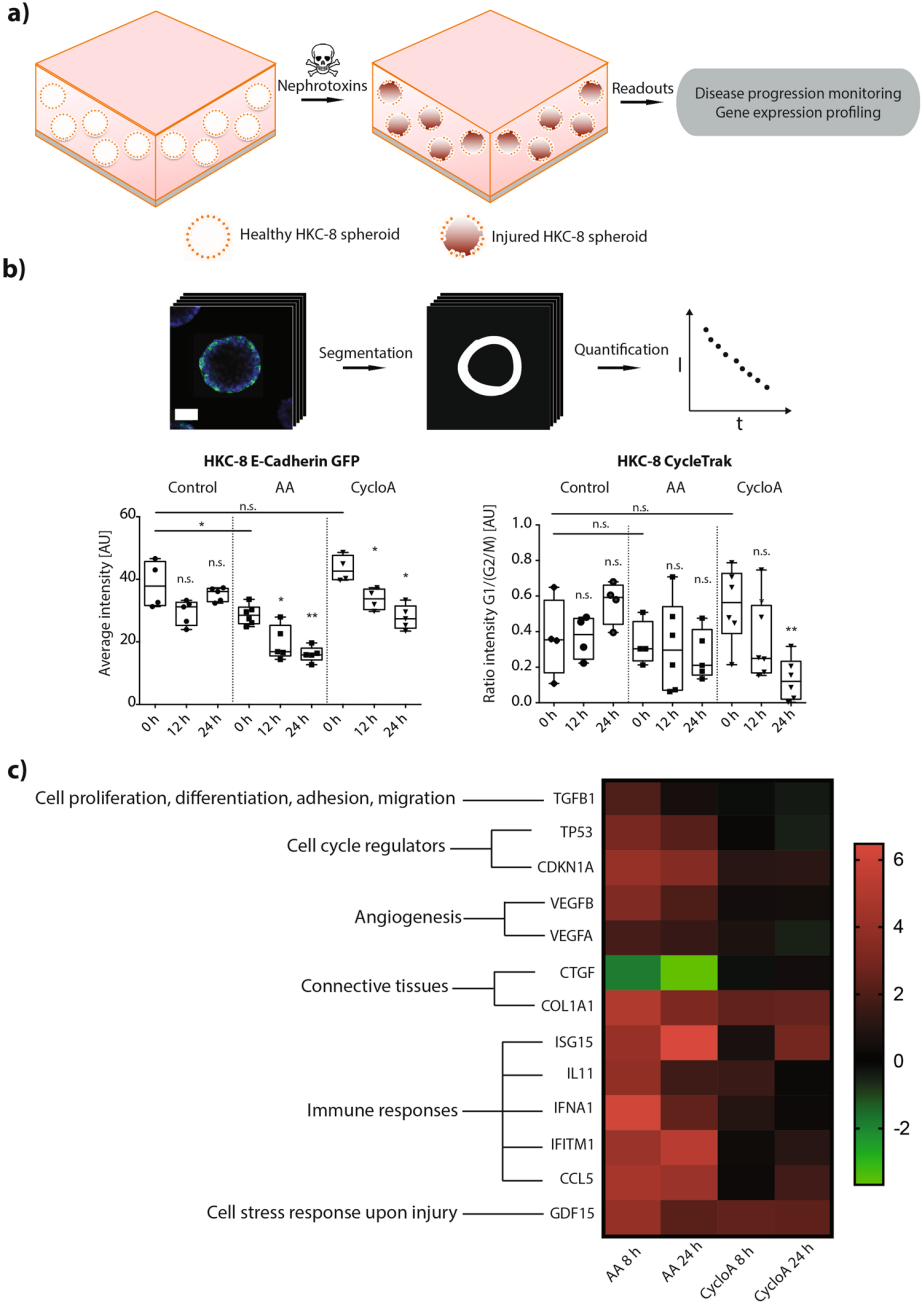
3D HKC-8 spheroids response to nephrotoxic compounds. (**a**) Schematic diagram of the HKC-8 spheroid response to nephrotoxins, (**b**) Quantitative image analysis of HKC-8 spheroids disease response by monitoring different reporter constructs (left, HKC-8 E-cadherin GFP; right, HKC-8 CycleTrak, n ≥ 4) (**c**) Kidney molecular tox gene panels consisting of ~1600 relevant genes analysis of the HKC-8 spheroids response to nephrotoxins (*P < 0.1, **P < 0.01; n.s., not significant (Mann Whitney *U* test). AA and cyclosporine A concentrations are 125 μM and 1.5 μM, respectively. For each group, spheroids were pooled from ~45,000 seeded cells. Scale bar, 30 μm (**b**).

To evaluate whether our *in vitro* confined microenvironment system can recapitulate the crosstalk reported between tubular epithelium and fibroblasts in the kidney *in vivo* ^33^, we exposed our tubular epithelial HKC-8 spheroids to clinically-relevant nephrotoxic drugs, such as cyclosporine A (CycloA)^34^, gentamicin^35^, diclofenac^36^, aristolochic acid (AA)^37^, and lithium chloride^38^ (Supplementary Figure S1). To characterize the epithelial cell injury with high temporal resolution, spheroids made of stably transfected fluorescent biosensors were adopted. Genetically-encoded fluorescent cell cycle (CycleTrak) and cell polarity (E-cadherin-GFP) reporters were used to characterize early sign of epithelial cell damage (Fig. 3a) as previously described^39–41^. Acquired volumetric spheroid images were subjected to a phenotypic profiling based on intensity, localization, and ratio of the reporter signals (see Materials and Methods, Fig. 3b, and Supplementary Figure S2). In parallel to the imaging effort, transcriptome analysis of a gene panel relevant to epithelial cell injury was performed (Fig. 3c and Supplementary Table T1).

We identified two nephrotoxic compounds, AA and CycloA, which induced a significant epithelial cell injury: epithelial cell polarity (*i*.*e*., E-cadherin GFP signal) decreased as early as 12 hours after both AA and CycloA administration (Fig. 3b). CycloA induced also an arrest of the spheroid epithelial cells in the G2/M phase 24 hours after toxic insult (Fig. 3b and Supplementary Figure S2). Overall, a mild reduction in spheroid viability was only noticeable 24 hours after CycloA administration (Supplementary Figure S3), indicating that the observed phenotypical changes at 12 and 24 hours originated from mostly unaffected cells. Notably, effects on cell polarity and cell cycle were more pronounced for CycloA than for AA.

Transcriptome analysis of nephrotoxin-treated spheroids showed upregulation of inflammatory genes (*e*.*g*., ISG15, IL11, IFNA1, IFITM1 and CCL5), angiogenesis-related genes (*e*.*g*., VEGFA and VEGFB), and transcripts coding for proteins known to increase epithelial cell ability to endure a hostile environment (*e*.*g*., GDF15) – and did so as early as 8 hours after drug administration (Fig. 3c). A pathway network analysis further revealed upregulation of genes associated with transmembrane transport of small molecules, transport of inorganic molecules, ABC-family proteins mediated transport, cellular responses to stress, interleukin-mediated signaling events, transforming growth factor β (TGFβ) receptor signaling, and lipid/lipoprotein metabolism (Supplementary Table T1). Analogous to the results obtained from optical imaging, CycloA treatment resulted in stronger gene signature modulation compared to AA treatment.

### Nephrotoxin-treated HKC-8 spheroids induce myofibroblast differentiation in the *in vitro* 3D co-culture system

**Figure 4:**
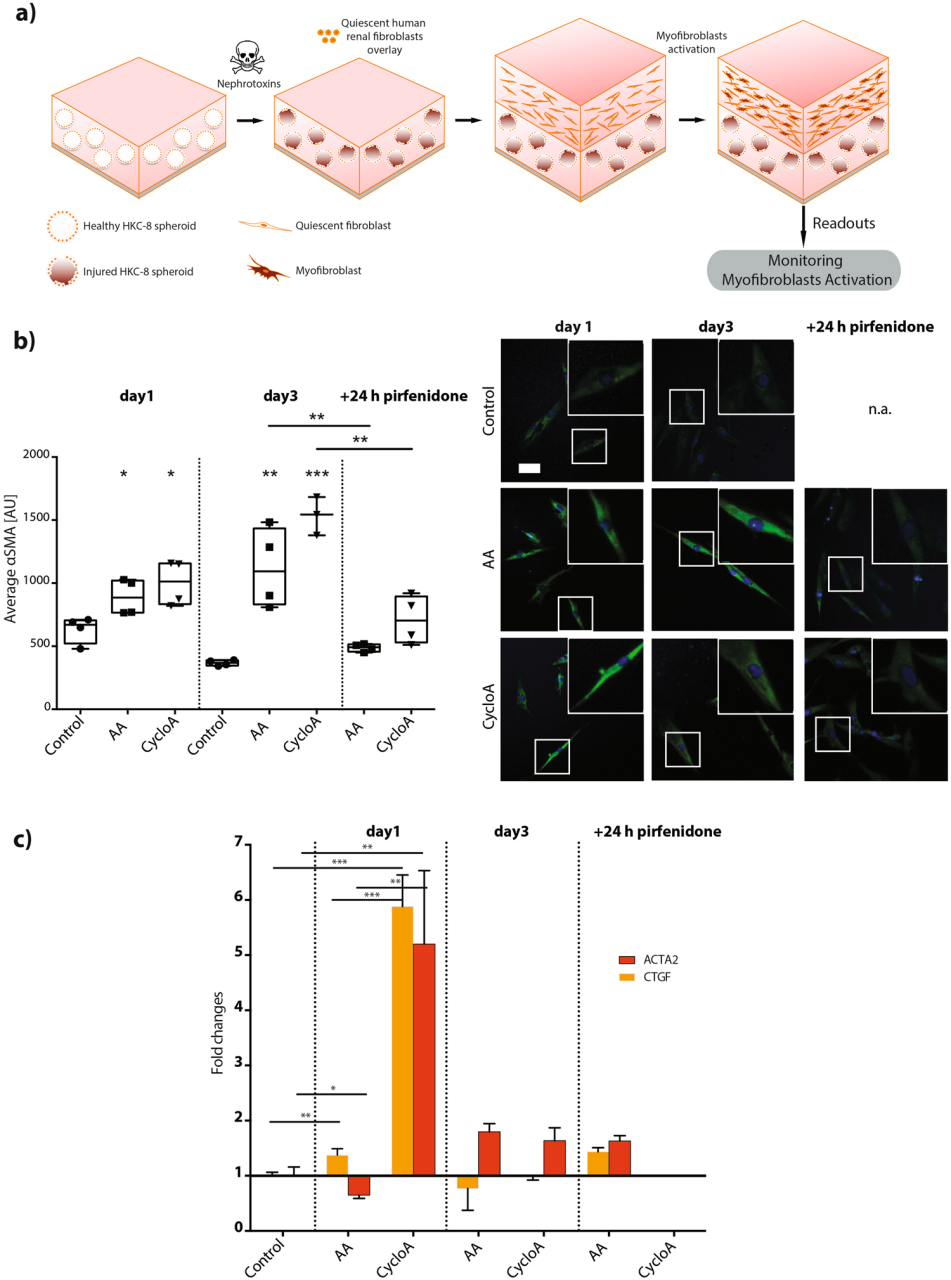
Image-based myofibroblast activation monitoring in the 3D co-culture setup. (**a**) Schematic diagram of the simulation of myofibroblast activation in the 3D *in vitro* co-culture setup through epithelial and mesenchymal crosstalk. (**b**) Activated myofibroblasts identification upon nephrotoxin treatment using αSMA immunofluorescent staining (n ≥ 3). (**c**) Myofibroblast signature genes validation with Taqman gene expression analysis (n = 4), *P < 0.1, **P < 0.01, ***P < 0.001 (two-tailed t test). AA and cyclosporine A concentrations are 125 μM and 1.5 μM, respectively. Scale bar, 5 μm (**b**).

We then aimed to investigate whether injured spheroids would modulate the phenotype of co-cultured fibroblasts. After 24 hours nephrotoxin treatment, epithelial spheroids were washed and overlaid with proliferating and migrating renal fibroblasts dispersed in a hydrogel matrix (Fig. 4a and Supplementary Figure S4), there by mimicking a simplified pathological microenvironmental interface between human kidney proximal tubular epithelial cells and renal fibroblast. Fibroblasts co-cultured with AA or CycloA insulted spheroids underwent dramatic morphological changes as early as 24 hours after seeding, acquiring expression of alpha-smooth muscle actin (αSMA^+^) (Fig. 4b). This transition was comparable to control fibroblasts that were monocultured in the same hydrogel matrix and treated with the pro-fibrogenic cytokine TGFβ1^42^ (Supplementary Figure S5). Fibroblasts co-cultured with undamaged HKC-8 spheroids maintained their quiescent phenotype. CycloA-treated HKC-8 spheroids induced stronger myofibroblast differentiation compared to AA-treated ones, which is in agreement with the stronger epithelial cell injury observed in the epithelial spheroid assay (Fig. 3).

Taqman gene expression assays of extracted co-cultured fibroblasts confirmed the stronger effect of CycloA in inducing myofibroblast activation. Both characteristic myofibroblast genes ACTA2 and CTGF, showed stronger upregulation in fibroblasts co-cultured on CycloA-treated HKC-8 spheroids than on AA treated ones (Fig. 4c). In both cases, we also detected upregulation of pro-fibrotic factors TGFβ1 and connective tissue growth factor (CTGF) - two key chemokines in modulating the tubulointerstitial microenvironment *in vivo* (Fig. 4c, Supplementary Figure S6)^29^.

### Pirfenidone treatment inhibits myofibroblast differentiation in the *in vitro* 3D co-culture system

The successful development of this comprehensive fibrosis model motivated us to test its capabilities in screening for anti-fibrogenic small molecule compounds. As a proof of concept, pirfenidone was selected based on its clinical relevance^43,44^ and its documented ability to inhibit TGFβ1-induced fibrogenesis^45,46^. Notably, as little as 24 hours pirfenidone treatment of the co-culture resulted in significantly decreased myofibroblast activation as shown by reduced amount of αSMA in fibroblasts (Fig. 4b). Likewise, the pronounced gene upregulation observed in the epithelial spheroids after CycloA treatment was counteracted by administration of pirfenidone (Fig. 4c, Supplementary Figure S6).

## Discussion

In the present work, we describe, characterize, and validate a novel *in vitro* 3D cell co-culture system aimed at high throughput screening for compounds capable of modulating the tubulo-interstitial microenvironment crosstalk. The sequential buildup of the 3D *in vitro* microenvironment enables both proper physiological cell differentiation and at the same time targeted pharmacological manipulation of epithelial cells before the introduction of fibroblasts. Although the key elements of the deteriorated renal microenvironment (i.e. the injured tubular epithelial cells and resident fibroblasts) are represented in the proposed *in vitro* co-cultured cellular system, the generated screening environment lacks several elements of the native environment, such as a native cellular matrix as well as a native basement membrane at the base of the spheroids. These structures place highly relevant constraints on the signaling that can occur between tubule and fibroblast cells^47^. The corollary of this limitation is that the crosstalk generated in the present *in vitro* culture system may not comprehensively reflect the pathophysiological process that takes place *in vivo*. Unfortunately, the use of native matrix, tried in the setup phase of the present study, introduced an unacceptable level of irreproducibility, a feature incompatible with a high throughput assay meant to identify *first-in-class* drugs. In spite of these limitations, the proposed co-cultured system allows exploring a totally new chemical space: compounds that can modulate fibrosis by impacting directly on the epithelial/mesenchymal cellular crosstalk rather than the mere fibroblast to myofibroblast transition^48^ or the epithelial to mesenchymal transition (EMT)^49^, thus capturing recently discovered biology^29–31^ in a single high throughput cellular assay.

In order to assess the pathological state of the epithelial spheroids, we combined 3D cell culture with fluorescent epithelial reporters, enabling time-lapse optical imaging of the early stages of epithelial cell injury exposed to nephrotoxic compounds^12,29,50^ followed by customized image-based analysis. Our phenotypic screening approach unveiled the precise temporal progression of cellular dynamics during early stage of epithelial damage that has previously been reported mainly *in vivo* ^40^. Gene expression profiling at relevant stages of disease progression unveiled additional genetic diversity and more refined insight into the origin and properties of relevant fibrotic responses. Moreover, we identified the optimal time window for introducing primary fibroblasts and for generating an effective pathological microenvironmental interface.

Using this refined disease model, we demonstrate that fully differentiated polarized tubular cell spheroids, when injured, induce activation of surrounding quiescent fibroblasts into myofibroblasts via modulation of a complex cellular crosstalk, the hallmark cellular phenotype characteristic of the fibrotic kidney^51,52^. Notably, our results further corroborate previous *in vivo* experiments^30,40^ showing that fibrosis can occur in the absence of a direct EMT process, since no sign of epithelial cell transformation (e.g. activation of S100A4 protein) was detected in injured epithelium (data not shown). The observed marked effect of CycloA in modulating the myofibroblast phenotype is well in line with results from calcineurin inhibitor treated patients who display a diseased pro-fibrotic epithelial phenotype^31^, characterized by the cytoplasmic accumulation of the E-Cadherin transcriptional repressor protein SNAI1^53^.

By inhibiting the TGFβ1 signaling pathway with a known fibrotic drug, we were able to interrupt fibrosis onset and rescue phenotypically healthy co-cultures, corroborating recent *in vivo* work that unveiled a key role of this chemokine in inducing diffuse fibrosis in the surrounding tubulo-interstitial space when locally overexpressed in renal tubules^22^.

Our results suggest that the present platform is well suited for imaging-based pharmacological drug screening of novel anti-fibrotic molecules that are capable of modulating the epithelial/mesenchymal crosstalk and whose relevance might apply well beyond the kidney to any other fibrotic disease such as pulmonary fibrosis^54^. The platform we describe here may also be used to assess potential side effects of antisense oligonucleotides, an emerging targeting modality characterized by a pronounced renal targeting and consequent renal toxicity^55^. To enable larger scale screenings by further increasing the throughput, parallelization of imaging and utilization of fast volumetric imaging modalities such as selective plane illumination microscopy (SPIM)^56,57^ can easily be implemented.

In order to allow for more refined patient-specific drug screens, the technology could likely be adapted to human induced pluripotent stem cell (hiPSC)-derived epithelial cell lines. Although the use of hiPSCs can provide refined insight into the genetically determined variability in drug susceptibility within a given population, hiPSC derived cells are usually harder to transfect with reporter transgenes and were therefore not considered for the present work. Advances in transgenesis of hiPSCs will make this approach even more relevant, eliminating the need for stable cell lines. Ultimately, combining the highly versatile imaging-based platform that we present here with novel biomarkers and fast volumetric imaging opens up the possibility to perform high throughput, high content screening of pharmacological compounds in a miniaturized 3D human fibrosis model.

## Methods

### Materials

Cellendes 3D Life Dextran hydrogel kit was purchased from Biocat Germany. Other chemical reagents were obtained from Sigma Aldrich, otherwise mentioned. The human kidney proximal tubular cell line (HKC-8) was obtained from University Hospital of Erlangen-Nuremberg Germany, and the human renal fibroblasts (PU009-F) were obtained from DV Biologics USA and maintained with the medium kit provided by the company. Some of the schematic drawings were in part prepared using a Toolkit-Suite from Motifolio Inc., USA.

### 3D co-culture setup

Human proximal tubular cells HKC8 (Johns Hopkins University, Baltimore, ML, USA) were cultured in DMEM/F12 media supplemented with 2.5% FBS, 1% Insulin-Transferrin-Selenium (ITS, BD 354352), Penicillin (100 Units/ml) and Streptomycin (100 µg/ml) at 37 °C and 5% CO_2_.

Prior to mixing with the single cell suspension, the dextran hydrogel kit was functionalized with RGD peptide following the kit protocol. The HKC-8 single cell suspension was mixed with the RGD-functionalized dextran hydrogel, crosslinked with PEG-based crosslinker provided in the kit and spotted as small droplets in the cell culture well plate. Notably, the overlaid co-cultured cells in the hydrogel are still amenable for imaging with an upright confocal microscope and/or a long-distance objective lens in inverted confocal microscope.

### HKC-8 spheroids characterization

To stain differentiation markers in HKC-8 spheroids such as β-Catenin, Pendrin, acetylated α-Tubulin, Glut1, and Aquaporin-1, HKC-8 spheroids in hydrogel were fixed in 4% paraformaldehyde for 30 minutes. The spheroids were then permeabilized with 0.1% Triton X-100 for 30 minutes, blocked with 1% BSA for 30 minutes, incubated with primary antibodies (Sigma Aldrich, Germany) and secondary antibodies (Life Technologies, Switzerland). The samples were then mounted with Fluorsave (Merck Chemicals, Germany) to minimize laser-induced photo bleaching. Microscopy images were acquired using a 40x water immersed objective lens (NA 1.2) on a Zeiss 710 upright and on a Zeiss 780 inverted confocal microscope, respectively. The 3D image stack was reconstructed using Imaris Software (Bitplane).

The molecular passive diffusion kinetics into the lumen of the spheroids were evaluated using fluorescent dextran compounds of two different molecular weights - one above the glomerular filtration molecular weight cutoff and another below (3 and 150 kDa, Life Technologies Switzerland), respective. To assess the compactness of cell-cell boundaries at the outermost layer of the HKC-8 spheroid, spheroids were imaged for 6 hours directly following the addition of the distinct dextran solutions.

Ultrastructural views of the spheroids were observed with a transmission electron microscope (TEM). HKC-8 spheroids in hydrogel were fixed in 4% paraformaldehyde for 1 hour, and further treated with 1% OsO_4_ for 2 hours at room temperature. The spheroids were subsequently dehydrated step-wise in ethanol (25%, 50%, 75%, 95% and 100%) for 10 minutes followed by 100% acetone twice for 20 minutes each. Upon dehydration, spheroids were then treated with 1:1 ratio mixture of acetone and araldite resin for 30 minutes at room temperature followed by overnight treatment at a 1:6 ratio at room temperature. On the following day, the samples were placed into araldite resin for 30 minutes at room temperature before transferring into a 40 °C oven for another 30 minutes. Araldite resin was subsequently changed followed by 1 hour treatments at 45 °C and 1 hour at 50 °C. Lastly, the samples were embedded with araldite resin at 60 °C for 24 hours. Sections of 90–100 μm thickness were sliced using a Leica EM UC 6 Ultramicrotome, collected onto 200-mesh copper grids and co-stained with uranyl acetate and lead citrate for 10 minutes each. Observation was undertaken with a Transmission Electron Microscope (TEM) (JEOL JEM-1010, Japan) at voltage 100 kV.

### Cell reporter line establishment and analysis

HKC-8 cells were transfected with E-cadherin GFP (Addgene) and or CycleTrak (Dr. Kulesa Laboraroty Stowers Institute for Medical Research Kansas City USA) plasmids through nucleofection (Lonza 4D-Nucleofector^TM^ System). Upon transfection, the cells were then expanded, sorted with FACS (Beckman Coulter MoFlo XDP), and maintained in the antibiotic resistance-containing medium.

HKC-8 E-cadherin GFP and HKC-8 CycleTrak clones were cultured in dextran hydrogel in an identical fashion to their parent clonal cell and followed the same ~12 day maturation time scale as non-transfected cells. On day 15 post-seeding, the spheroids were incubated with two nephrotoxins, aristolochic acid and cyclosporine A at concentrations 125 μM and 1.5 μM, respectively. These spheroids were imaged using confocal microscopy every 12 hours. The images were analyzed using a custom MATLAB (Mathworks) script, which i) automatically detects the outline of the spheroids based on ubiquitous nuclei staining and fluorescent reporter signal, ii) segments the hollow rim with consistent thickness across samples, and iii) quantifies reporter fluorescence on the segmented area.

### Ampliseq

HKC-8 spheroids were treated with the selected nephrotoxins at concentrations 125 μM for aristolochic acid, 1.5 μM for cyclosporine A. These spheroids were lysed with RLT buffer at 8 and 24 hours upon treatment. The RNA was extracted with (RNA Isolation Kit Qiagen) and run in AmpliSeq gene array with ~1600 genes relevant with molecular toxicology (Thermofisher Scientific). For each probe on the chip, average expression levels were obtained, and the derived changes plus their statistical significance were calculated. Reads were aligned to the human transcriptome (hg19) and mapped to the 997 pathway reporters using the TMAP algorithm. Normalization and detection of differentially expressed genes in between the samples was done using the TMM algorithm and the exact test within the edgeR bioconductor package. Pathway reporters were considered statistically significant with a FDR < 0.05 and expression levels > 5 counts per million.

Fold changes between the control sample and the corresponding treatment condition were calculated and a heat map was created. Using the complete, ranked list of calculated changes, Gene Set Enrichment Analyses (GSEA) was run on gene sets provided by REACTOME and other sources through the Pathway Commons collection (www.pathwaycommons.org)^58^. For each such set, a significance level (technically, an FDR-corrected q-value) was calculated to indicate the joint up- or down-regulation of its member genes. The collection contains around 1,600 gene sets, which are partially overlapping and redundant. The readouts are then normalized to the readouts of control samples.

### Myofibroblast immunofluorescent staining

Myofibroblasts appearance in the overlaid human renal fibroblast population was characterized by immunofluorescent staining of α smooth muscle actin (αSMA). The proliferation of the human renal fibroblasts was observed by performing a PicoGreen Assay (Life Technologies, USA). The overlaid fibroblasts in hydrogel were fixed in 4% paraformaldehyde for 30 minutes, permeabilized with 0.1% Triton X-100 for 30 minutes, blocked with 1% BSA for 30 minutes, incubated with primary antibodies (Sigma Aldrich, Germany) and secondary antibodies (Life Technologies, Switzerland). The samples were then mounted with Fluorsave (Merck Chemicals, Germany) to minimize laser-induced photo bleaching. Microscopy images were acquired with 40x objective lens on a Zeiss Meta 710 upright confocal microscope. The maximum intensity projection images were then quantified and analyzed against the untreated sample. Pirfenidone as the chosen drug model to inhibit myofibroblasts proliferation was added after the fibroblasts were activated for at least a day.

### Gene expression array analysis (Fluidigm)

Human renal fibroblast upper layers were gently separated from the bottom hydrogel, washed once with PBS and lysed with 350 µl RLT buffer. RNA was extracted from cells using an RNeasy 96 Kit (Qiagen, 74181) according to the manufacturer’s instructions (RNeasy 96 protocol for Isolation of total RNA from animal cells, using spin technology). cDNA was synthesized with Transcriptor First Strand cDNA Synthesis Kit from Roche (04 897 030 001). RNA was mixed with oligo(dT)_18_ Primer 2.5 µM and random hexamer primer 60 µM, and denatured by heating to 65 °C for 10 minutes. Afterwards, 1x reaction buffer (8 mM MgCl2), 20U RNase Inhibitor, 1 mM dNTP and 10U Transcriptor reverse transcriptase were added and the synthesis reaction was performed for 10 minutes at 25 °C, followed by 50 °C for 60 minutes and stopped by heating at 85 °C for 5 minutes.

QPCR reactions were performed in 48-well Fluidigm chip for relative cDNA quantification for genes of interest using LC480 Probes Master (Roche Applied Science) and specific TaqMan Probes and Primers (Applied Biosystems), according to the manufacturer’s instructions. Analysis was conducted using the Δ-Δcycle threshold (Ct) method, which determined fold changes in gene expression relative to a control-treated sample. Each analysis reaction was performed in duplicate, with 3 samples per condition. Gene expression was normalized to nonsense mediated mRNA decay factor (HBMS) and ubiquitin C (UBC), which were used as internal reference genes and was measured with the LightCycler 480 Instrument (Roche Applied Science).

Catalogue numbers for primers and probes: Alpha smooth muscle actin 2; ACTA2; Hs00426835_g1. Collagen, type I, alpha 1; Col1A1, Hs00164004_m1. Collagen, type III, alpha 1; Col3A1, Hs00943809_m1. Connective tissue growth factor; CTGF; Hs00170014_m1. Nonsense mediated mRNA decay factor; HBMS; Hs01089943_m1. Inhibitor of DNA binding 1; ID1; Hs03676575_s1. Transforming growth factor, beta 1; TGFβ1; Hs00998133_m1. Ubiquitin c; UBC; Hs01871556_s1.

### Code availability

The custom Matlab script is available upon request.

### Data availability

The detailed datasets generated during and/or analyzed during the current study are available from the corresponding authors upon request.

## Electronic supplementary material

Supplementary Information

Table T1
