# Supplementary material for: Monitoring and manipulating cellular crosstalk during kidney fibrosis inside a 3D in vitro co-culture
Source: Sci Rep. 2017 Nov 3;7:14490. doi: 10.1038/s41598-017-12683-y (PMC5670242; doi:10.1038/s41598-017-12683-y)
Supplement: Supplementary file 1 — Supplementary Information [file 41598_2017_12683_MOESM1_ESM.pdf]

Supplementary Information

Monitoring and manipulating cellular crosstalk during kidney fibrosis inside a 3D in vitro co-culture

Bramasta Nugraha[1,2,†], Manuel A. Mohr[1], Aaron Ponti[1], Maximilian Y. Emmert[3-6], Franziska Weibel[2], Simon P. Hoerstrup[3-6], Solange Moll[7], Ulrich Certa[2], Marco Prunotto[2,‡], Periklis Pantazis[1,‡]

This file includes

Supplementary Figure 1.	List of clinically relevant nephrotoxins cytotoxicity study in HKC-8 spheroids (24 hours)
Supplementary Figure 2.	Representative HKC-8 spheroid reporter images during nephrotoxin treatment
Supplementary Figure 3.	Toxicity assessments of aristolochic acid and cyclosporine A
Supplementary Figure 4.	Human renal fibroblasts 3D culture in dextran hydrogel
Supplementary Figure 5.	TGFβ1-treated fibroblasts monoculture identification using αSMA-staining
Supplementary Figure 6.	Myofibroblasts gene expression analysis using the Taqman gene expression assay
Supplementary Table 1.	Kidney molecular toxicity pathway analysis of the HKC-8 spheroids’ response to nephrotoxins

Supplementary Figures

Drugs compound	Pathological results	Working concentration
Cyclosporine A	Tubular atrophy, collagen matrix deposition in interstitial space	1.5 μM for 24 h incubation
Gentamicin	Tubular necrosis, proximal tubule desquamation	No response
Diclofenac	Proximal tubule apoptosis, damaged and hyperthrophied glomerulus	No response
Aristolochic acid	Acute tubular necrosis, tubulointerstitial damage	125 μM for 24 h incubation
Lithium	Tubular distrophy, interstitial fibrosis	No response

Supplementary Figure S1. List of clinically relevant nephrotoxins cytotoxicity study in HKC-8 spheroids (24 hours)

HKC-8 spheroids' responses to various clinically relevant nephrotoxins were examined by the cell viability assay (MTT). Of the five tested nephrotoxins, HKC-8 spheroids showed a dose dependency to cyclosporine A and aristolochic acid. Gentamicin, diclofenac, and lithium did not induce an appreciable cytotoxic effect on the HKC-8 3D model. This discrepancy is probably due to the fact that we employed immortalized, stably-transfected fluorescent cell lines for the 3D model, which might have changed genetically under the subjected experimental conditions, leading to the phenotypic differences.

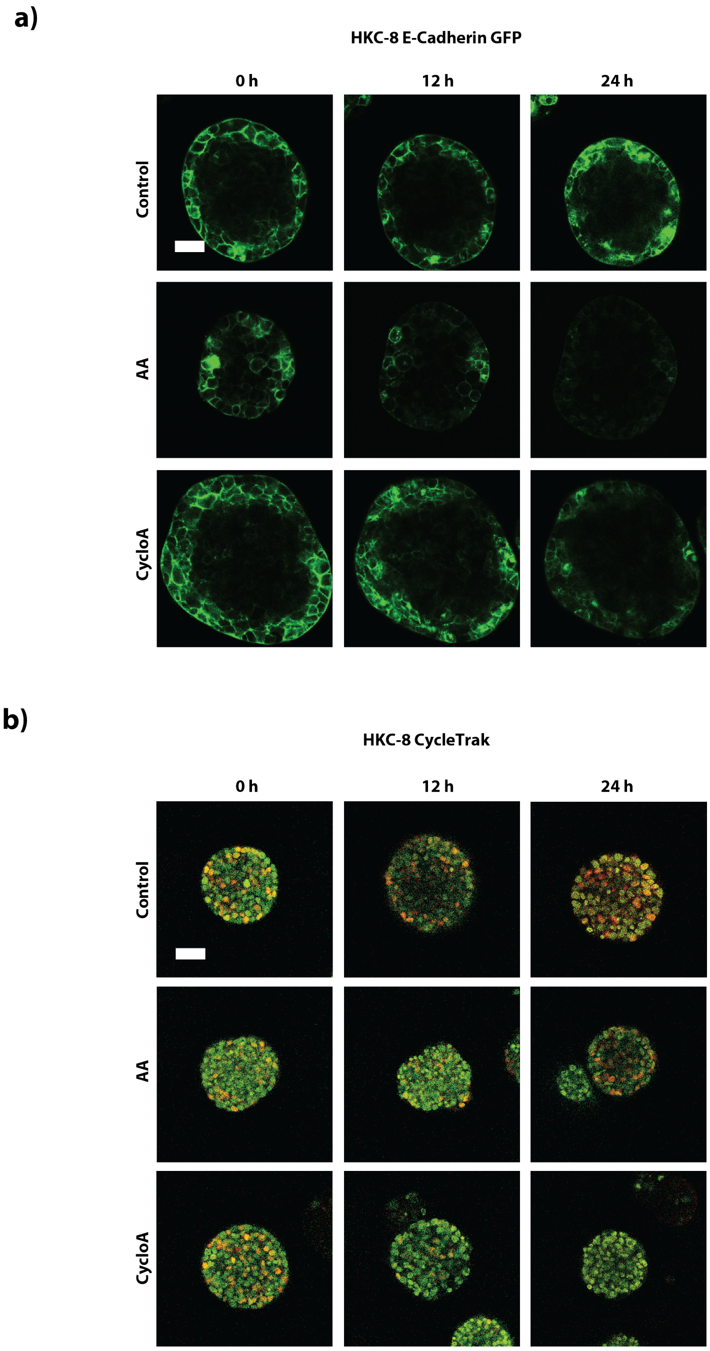

Supplementary Figure S2. Representative HKC-8 spheroid reporter images during nephrotoxin treatment

(**a**) HKC-8 E-cadherin GFP and (**b**) HKC-8 CycleTrak spheroids during nephrotoxins treatment. (**a**), green signal represents E-cadherin. (**b**), green signal represents G2/M phase, red signal represents G1 phase, and yellow signal represents S phase. Scale bar, 50 µm.

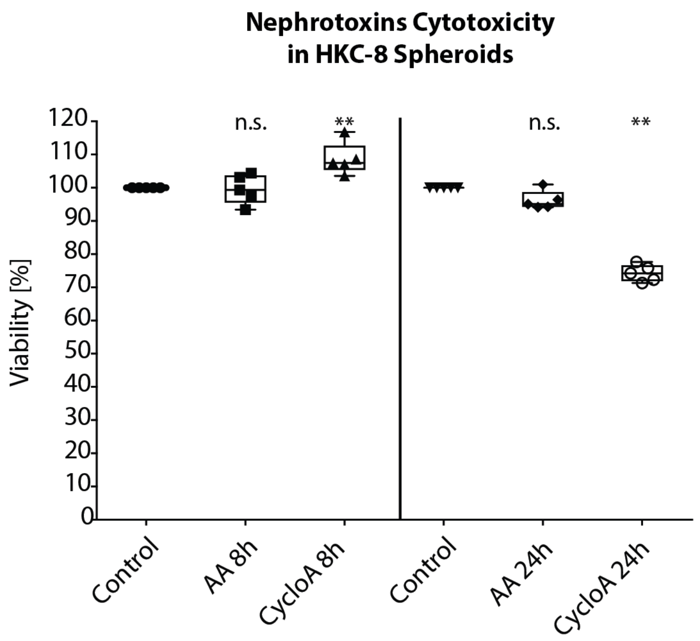

Supplementary Figure S3. Toxicity assessments of aristolochic acid and cyclosporine A

Cytotoxicity of aristolochic acid and cyclosporine A at the working concentration in HKC-8 spheroids were examined by the cell viability assay (MTT) after 8 and 24 hours. After 24 hours of drug treatment, only cyclosporine A treatment induced noticeable viability reduction (n=5). **$P<0.01$, n.s., not significant (Mann Whitney *U* test).

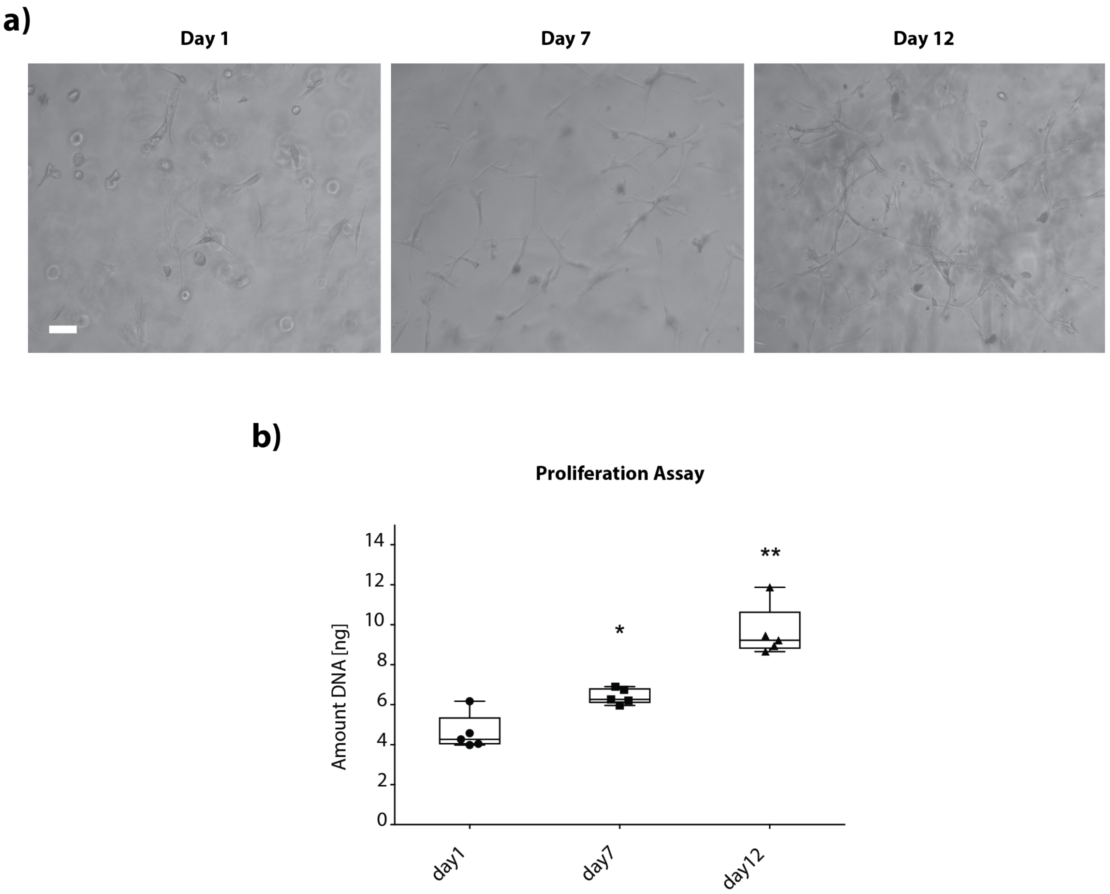

Supplementary Figure S4. Human renal fibroblasts 3D culture in dextran hydrogel

Cell migration and proliferation were investigated using (**a**) bright field images and (**b**) the PicoGreen Assay, respectively. Proliferating renal fibroblasts migrated in the hydrogel over the culture forming sprouting constructs (n=5). *P<0.1; **P<0.01 (Mann Whitney *U* test). Scale bar, 20 µm (**a**).

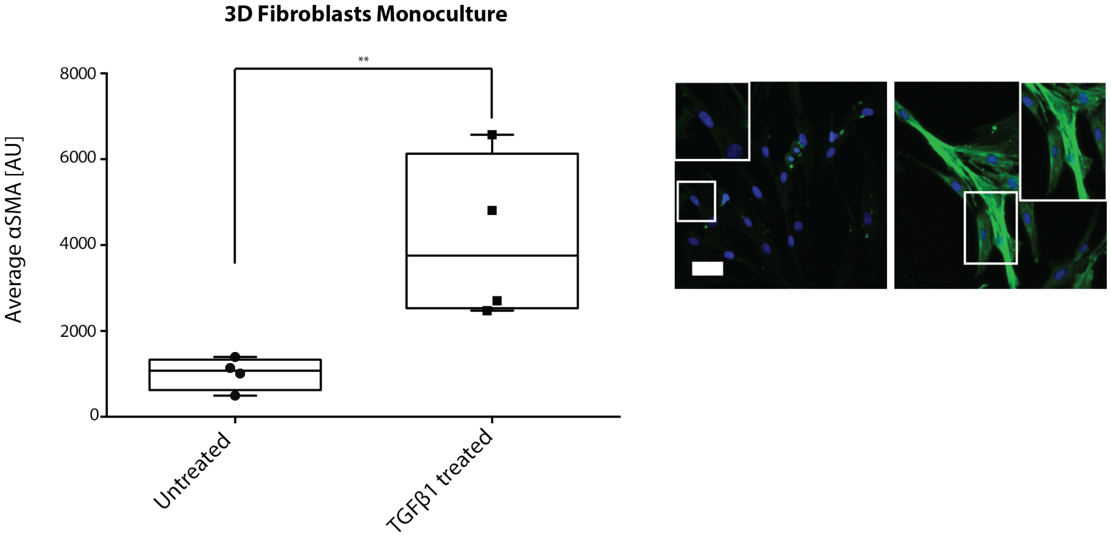

Supplementary Figure S5. TGFβ1-treated fibroblasts monoculture identification using αSMA-staining

To confirm that the human renal fibroblasts used in this work respond to profibrogenic cytokine TGFβ1, a 3D monoculture of human renal fibroblasts in the dextran hydrogel containing MMP-sensitive crosslinker was treated with 10 ng/mL TGFβ1 for 3 days. The cells were then fixed and stained for αSMA (n=4). **P<0.01. Scale bar, 5 μm.

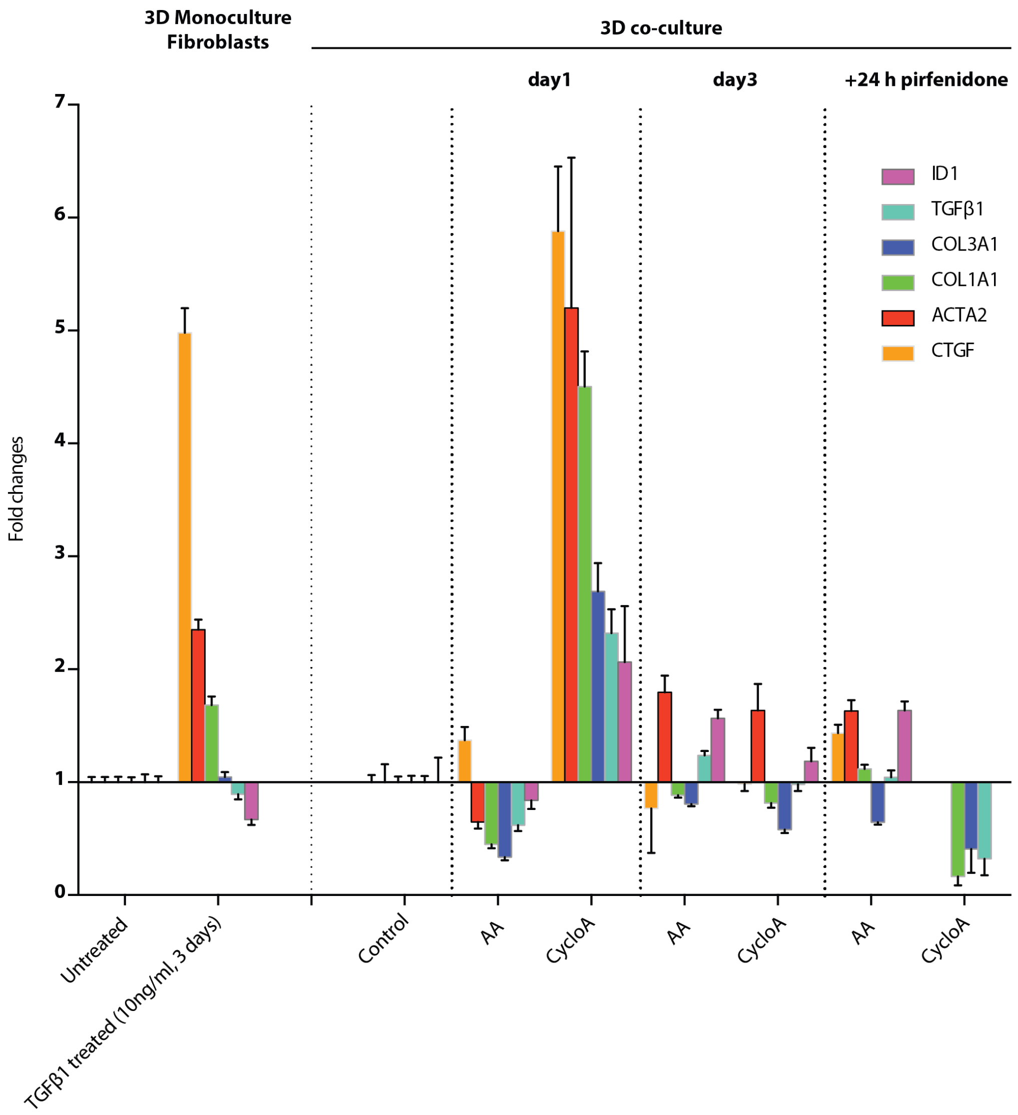

Supplementary Figure S6. Myofibroblasts gene expression analysis using the Taqman gene expression assay

The signature genes of myofibroblasts were analyzed using the Taqman assay, in comparison to the human renal fibroblasts treated with TGFβ1 as control. Myofibroblasts activation is inhibited upon addition of pirfenidone, a known inhibitor of TGFβ1 production (n=4).

Supplementary Table T1. Kidney molecular toxicity pathway analysis of the HKC-8 spheroids' response to nephrotoxins

At 8 and 24 hours after treatment of 125 µM aristolochic acid and 1.5 µM cyclosporine A, the RNA was extracted from HKC-8 spheroids and analyzed with Mol Tox panel genes (~1600 genes) in Ampliseq. The count readouts from these genes were classified into relevant pathways/mechanisms. The readouts were then normalized to the readouts of control samples.
